# Which role models are effective for which students? A systematic review and four recommendations for maximizing the effectiveness of role models in STEM

**DOI:** 10.1186/s40594-021-00315-x

**Published:** 2021-12-02

**Authors:** Jessica R. Gladstone, Andrei Cimpian

**Affiliations:** 1 Department of Psychology, New York University, 6 Washington Place, New York, NY 10003 USA; 2 Present Address: Department of Foundations of Education, Virginia Commonwealth University, 1015 West Main Street, Richmond, VA 23284 USA

**Keywords:** Role models, Motivation, Science, STEM, Diversity

## Abstract

Is exposing students to role models an effective tool for diversifying science, technology, engineering, and mathematics (STEM)? So far, the evidence for this claim is mixed. Here, we set out to identify systematic sources of variability in STEM role models’ effects on student motivation: If we determine *which role models* are effective for *which students*, we will be in a better position to maximize role models’ impact as a tool for diversifying STEM. A systematic narrative review of the literature (55 articles) investigated the effects of role models on students’ STEM motivation as a function of several key features of the role models (their perceived competence, their perceived similarity to students, and the perceived attainability of their success) and the students (their gender, race/ethnicity, age, and identification with STEM). We conclude with four concrete recommendations for ensuring that STEM role models are motivating for students of all backgrounds and demographics—an important step toward diversifying STEM.

**Supplementary Information:**

The online version contains supplementary material available at 10.1186/s40594-021-00315-x.

## Introduction

In the United States, STEM fields fail to attract and retain women and racial/ethnic minorities in numbers proportional to their share of the population (e.g., National Science Foundation, 2017). Concerned about this inequity, many researchers, teachers, and policymakers have been seeking ways to increase the gender and racial/ethnic diversity of the STEM workforce. A popular tool for this purpose is to introduce students to STEM role models, which we define here as individuals who can positively shape a student’s motivation by acting as a successful exemplar. Well captured by the slogan “you can’t be what you can’t see” (variously attributed to Gloria Steinem, Sally Ride, and Marian Wright Edelman, founder of the Children’s Defense Fund), the use of role models is often billed as the one-stop solution for increasing diversity in STEM (e.g., Dean, 2014; Levere, 2018). And role models seem like a promising tool indeed—for instance, exposure to role models is relatively low-cost (Herrmann et al., 2016) and is flexible and adaptable to a wide range of contexts and student ages (Lawner et al., 2019).

However, the empirical evidence for the effectiveness of role models as a means of motivating diverse students to pursue STEM is sparse and not altogether straightforward. For example, a 2007 practice guide released by the Institute of Education Sciences on means to encourage girls in math and science concluded that there is a “low” level of empirical evidence for the recommendation to expose girls to role models (Halpern et al., 2007). Although this guide was released more than a decade ago, the empirical evidence for role models as a means of increasing diversity in STEM remains complex and contradictory (e.g., Lawner et al., 2019). Yet, these nuances have not blunted excitement among educators and the general public about role models as a motivational tool—if anything, enthusiasm for role models has probably increased in the intervening time.

Thus, our goal here was to provide an accessible and brief, but at the same time systematic (Siddaway et al., 2019), review of the current literature on role models to assess their effectiveness as a motivational tool for diversifying STEM. However, rather than focusing on the general question of whether role models can motivate diverse groups of students (the answer to which likely depends on many specifics), we set out to answer a more targeted set of questions about which role models are effective for which students in STEM. Specifically, we examined two types of potential moderators of role models’ effectiveness: (1) the features of the role models themselves (which we term “role model moderators”), and (2) the characteristics of the students exposed to the role models (which we term “student moderators”). We briefly unpack each of these categories of moderators before describing the theoretical frameworks that anchored this research.

With respect to role model moderators, our systematic review aimed to identify which features of a role model are effective in motivating STEM pursuits and, conversely, which features of a role model backfire, lowering student motivation. We used prominent theories of motivation to identify the features of role models that might be expected a priori (on theoretical grounds) to influence their effectiveness as motivational tools. Specifically, we focused on social cognitive theory (Bandura, 1977, 1986, 1997), expectancy–value theory (Eccles & Wigfield, 2020), mindset theory (Dweck, 1999, 2006; Dweck & Leggett, 1988), and attribution theory (Graham, 2020; Weiner, 1985) and drew out each theory’s implications for the features a role model should have to be effective. We then assessed the level of evidence in the literature for each of these theoretically-motivated role model moderators.

With respect to student moderators, our systematic review investigated how students’ gender and race/ethnicity moderated the effects of role models, consistent with the focus of our review on diversifying STEM. In addition, we investigated whether role models vary in their effectiveness as a function of student age, from elementary school to college. The final student moderator that was considered here was students’ attitudes toward STEM: Students who have not yet identified with this domain (which is likely true of many students from underrepresented backgrounds) may have different concerns, and as a result, may be motivated by different types of role models, than students who are farther along in their STEM educational trajectory (Drury et al., 2011). In other words, the qualities of a role model that can help attract a student to STEM in the first place and make them more likely to identify with this domain may be different from the qualities of a role model that can help retain a student and solidify their identification with STEM.

To maximize the usefulness of this systematic review for the education research and practice community, we conclude with a set of concrete recommendations extracted from our review of the literature. These recommendations are intended to increase the effectiveness of role-model interventions as a tool for diversifying STEM. We hope these guidelines will be useful not just for researchers and policymakers who are considering large-scale interventions that involve role models but also for educators who might want to use role models in their classrooms.

### Defining role models

Above, we defined role models as individuals who can positively shape a student’s motivation by acting as a successful exemplar. To arrive at a more precise definition, it is helpful to further differentiate role models from mentors and sponsors (e.g., Crosby, 1999; Downing et al., 2005; Gibson, 2004). Although all three types of career guides can be helpful to students, role models are distinguished from the other two types of career guides by the fact that they need not (and in fact often do not) have any prior relationship with the students whom they are influencing. In contrast, mentors and sponsors must have a prior relationship with the students to qualify as such, with the difference between them being that mentors provide counseling and general encouragement, whereas sponsors provide practical guidance (e.g., Downing et al., 2005). Thus, for purposes of our systematic review, we focused exclusively on empirical evidence concerning the motivational effects of exposure to STEM experts with whom students have no prior relationship. This distinction ensures that the conclusions we draw from our systematic review pertain to role models per se rather than capturing a more diffuse mix of role models, mentors, and sponsors.

### Role model moderators: What features do effective role models possess?

To inform our systematic review, we look to four well-established theories of motivation that have direct implications for the features that a role model should embody to be motivating. We briefly summarize each theory before drawing out its implications with respect to role models. To clarify, our analysis of these theories is not part of the systematic review per se—it simply lays the conceptual groundwork for the systematic review of the empirical evidence, which provided a test of the features highlighted here. It is also important to note that we are not the first to identify these role model features. Inspired in part by the same theoretical frameworks we integrate here, previous studies have explored how some of these features (e.g., a role model’s competence; Marx & Roman, 2002) relate to students’ motivation. Thus, the contribution of the present work does not lie in identifying the role model moderators per se but rather in systematically reviewing the evidence for them, which to our knowledge has never been done. The section below simply provides a theoretical anchor for the systematic review that follows. One final caveat: In this section, we discuss which features make role models motivating in a general sense, without factoring in whether these motivational effects vary as a function of students’ own characteristics; the latter issue is tackled in the section on student moderators.

#### Social cognitive theory

At the heart of Bandura’s social cognitive theory (Bandura, 1977, 1986, 1997) is the motivational construct of self-efficacy—one’s perceived ability to learn and do well in a domain. There is a large and growing body of work that demonstrates that self-efficacy is an important predictor of various student outcomes, including students’ choice of activities, their persistence in a task or domain, and their achievement (Bandura, 1997; Klassen & Usher, 2010; Schunk & Usher, 2019). Another central tenet of social cognitive theory is that students’ self-efficacy is shaped by their social environments (Schunk & Usher, 2019) and that engaging in observational learning is part of the process by which students’ own self-efficacy develops (Schunk & DiBenedetto, 2016, 2020).

Social cognitive theory’s connection to role models is straightforward. In fact, Bandura (e.g., 2001) explicitly stated that a key source of self-efficacy is observing a relatable role model succeed on a similar task. Specifically, there are at least three features of the role model that can affect students’ self-efficacy and achievement (Bandura & Walters, 1963; Schunk & DiBenedetto, 2020). The first feature is the perceived competence of the role model^1^: Students who observe others perform successfully on a task may believe that they can also be successful on the task. In other words, students may believe they can emulate a role model’s performance, which then raises their own self-efficacy (Schunk & DiBenedetto, 2020; Schunk & Usher, 2019). The second feature is the perceived similarity (or relatability) of the role model to the self (i.e., the student). According to Schunk and Usher (2019), students who perceive the role model to be similar to themselves are more likely to be influenced by the model. This is because sharing a degree of similarity makes the role model’s success more informative about the students’ own chances of succeeding in the future (Bandura, 1986; Schunk & DiBenedetto, 2020). Third, students’ self-efficacy should benefit to the extent that they perceive the role model’s success to be attainable. If what the role model has achieved feels out of reach for a student, their self-efficacy in the relevant domain may suffer.

The third feature (perceived attainability) is related to the second (perceived similarity) insofar as the success of a model who is perceived as similar to the self may thereby also be perceived as more attainable. However, a role model’s success can be perceived as attainable for other reasons as well (e.g., the steps to achieve it are clear), regardless of the student’s similarity to the model. The first feature we discussed (perceived competence) has implications for attainability as well. Although portraying the role model as successful may inspire a student to believe that they can succeed as well, an extraordinary level of success might feel unattainable and therefore be demotivating (e.g., “she can do that, but I can’t!”).

In summary, social cognitive theory suggests that role models can increase students’ self-efficacy, an important motivational variable. Extrapolating from this theory of motivation, we argue that the features of a role model that are key for students’ self-efficacy are the perceived competence of the role model, the perceived similarity to the role model, and the perceived attainability of the role model’s success.

#### Expectancy–value theory

According to expectancy–value theory, newly labeled situated expectancy–value theory (Eccles & Wigfield, 2020), students’ motivation to pursue an activity is a function of students’ beliefs about the likelihood of success in that activity (“expectancy”) and the value they perceive that activity to have for them (“value”) (Eccles & Wigfield, 2002, 2020). Consistent with this claim, students’ expectancies for success in a domain (e.g., STEM) and the subjective value they attach to that domain are strong predictors of outcomes such as achievement, course enrollment intentions, and college aspirations (e.g., Lauermann et al., 2017; Nagengast et al., 2011; Perez et al., 2014). Relevant for our purposes here, expectancy–value theory proposes that students’ expectancies for success and subjective task values are shaped by various socialization factors, including role models (Eccles & Wigfield, 2020; Parsons et al., 1982; Simpkins et al., 2015; see also Morgenroth et al., 2015). In what follows, we extrapolate from expectancy–value theorizing the features that should make a role model motivating, focusing in turn on expectancies for success and subjective values.

With respect to expectancies, the arguments that we made in the context of social cognitive theory apply here as well. This is so because students’ self-efficacy in a domain shapes their expectancies for success in that domain (see Wigfield & Eccles, 2000, for some nuances); thus, the features of a model that increase students’ self-efficacy should increase their perceived likelihood of success as well. With respect to subjective values, we argue that the model’s similarity to the self may be particularly effective in boosting the value that students assign to a particular domain. If students see someone who is meaningfully *like them* pursue a STEM career, for example, they may infer that STEM careers are a good fit for people like them—that these careers allow people like them to pursue goals that are important to them and express core aspects of their selves. Such inferences should increase the value of a STEM career in students’ eyes.^2^ In addition, these inferences seem particularly likely if students perceive the role model to be similar at a deeper, psychological level (“who they are as a person”)—not merely in terms of surface-level demographic characteristics (“what they look like”).

In summary, although expectancy–value theory and social cognitive theory focus on different aspects of motivation, they converge on similar desirable features in a role model: Extrapolating from expectancy–value theory, we argue that the features of a role model that are key for boosting students’ expectancies for success in STEM and their STEM subjective value are the perceived competence of the role model, students’ perceived similarity to the model, and the perceived attainability of the model’s success. A role model’s ability to boost the subjective value of STEM may depend in particular on their psychological (vs. merely demographic) similarity to the student, which highlights STEM as a good match with students’ deeper selves/identities and with their long-term goals and aspirations.

#### Mindset theory

The term “mindsets” refers to students’ beliefs about the malleability (vs. fixedness) of ability in a domain (e.g., Dweck, 1999, 2006; Dweck & Leggett, 1988). Specifically, a growth mindset is the belief that ability in a domain can be developed over time, whereas a fixed mindset is the belief that ability is fixed and stable over time. Growth mindsets put students in the “driver’s seat”—they give students a sense of control over their outcomes and promote the belief that success is attainable regardless of one’s current ability level, which in turn emboldens students to seek challenges and use setbacks as opportunities to learn (e.g., Blackwell et al., 2007; Cimpian et al., 2007; Mueller & Dweck, 1998; Porter et al., 2022; Yeager et al., 2019).

Students’ mindsets are shaped by messages from those around them, such as teachers and parents (e.g., Cimpian et al., 2007; Gunderson et al., 2013). Extrapolating from mindset theory, we argue that role models are motivating when they project the message that the abilities needed for success in STEM can be developed (rather than being fixed quantities that only some students possess), which should foster a growth mindset toward this domain. Because the upshot of these growth-oriented messages is to make success appear within reach for all students, we classify this feature of a role model under the broader category of perceived attainability. Although a role model’s STEM success may seem attainable for other reasons as well, the role model’s growth-oriented messages (e.g., that they have failed and learned from it) should be powerful motivators from the viewpoint of mindset theory.

#### Attribution theory

The predictions of mindset theory with respect to role models are aligned with those of another classic framework for understanding motivation: attribution theory (Graham, 2020; Weiner, 1985). According to attribution theory, student motivation is influenced by how students explain the causes of success and failure in a domain, with certain causal attributions being particularly likely to maintain or even increase motivation and others decreasing motivation instead (for a recent review, see Graham, 2020).

Concerning role models, the key question from the perspective of attribution theory is this: How do students explain the role model’s success in STEM? (Or, as the case may be, how does the role model explain their own success to students?) Some attributions might make the role model’s success seem more attainable than others and might thereby boost students’ own motivation to pursue this domain. Extrapolating from attribution theory, we argue that attributions to internal, unstable, and controllable factors (e.g., effort) are most likely to convey to students that they too can achieve success in STEM. This combination of causal features is necessary, we claim, for a role model to be effective from the viewpoint of attribution theory. If the role model’s success is instead portrayed as being due to, say, external factors (e.g., luck) or factors beyond the student’s control (e.g., innate ability), many students may justifiably doubt that they can duplicate the role model’s success, which would, in turn, undermine their motivation. Given that the recommendation we derived from mindset theory also promotes a focus on an internal, unstable, and controllable attribute (i.e., malleable STEM ability), the two theories are largely in agreement about what makes a role model effective.^3^

#### Interim summary

Our analysis of four theories of motivation suggested several features of a role model that should be motivating to students. Specifically, a role model’s perceived competence, their perceived similarity to the student, and the seeming attainability of their STEM career should each increase students’ own motivation to pursue STEM. We used these features to structure our systematic review of the literature on role models, assessing the level of evidence for each feature.

### Student moderators: Do student characteristics moderate the effect of role models?

In keeping with the present focus on role models as a tool for diversifying STEM, our systematic review first examined students’ gender and race/ethnicity as potential moderators of role models’ effectiveness; these two dimensions are particularly relevant to efforts to diversify STEM (e.g., Leslie, Cimpian, et al., 2015). Several patterns of results are possible. We might find, for example, that the features identified in the preceding section make a role model equally motivating for all students, regardless of their gender and race/ethnicity. In this case, role models could still serve as a tool for making STEM more diverse since they may bring underrepresented-group students closer to the threshold where they could envision a career in these fields. Alternatively, these features might be differentially effective in motivating underrepresented- and majority-group students, in part because the experiences of these groups in STEM contexts are different as well—for instance, students from underrepresented groups feel less welcome and supported in these contexts (e.g., Boston & Cimpian, 2018; Cheryan et al., 2015; Chestnut et al., 2018). Of course, it is also possible that exposure to role models could backfire, among students more generally or among underrepresented-group students specifically. The latter possibility is more concerning and perhaps also more likely, since students from underrepresented groups may more often perceive STEM role models as dissimilar to themselves and pursuit of these careers as beyond their reach.

In addition to gender and race/ethnicity, we also considered student age as a moderator of role models’ effects. Since STEM-related motivational variables undergo considerable change over the course of childhood and adolescence (e.g., Weidinger et al., 2017), as do students’ more general achievement-related beliefs and attitudes (e.g., Butler, 2005; Cimpian, 2017), it is important to examine the effectiveness of role models across a broad age range. Such an examination can potentially answer a range of fundamental questions about role models as a motivational tool: What is the earliest age at which role models have been documented to be effective? Does their overall effectiveness vary with student age? Do role model features vary in their importance with student age? If so, which features are most motivating for students of various ages?

The final student moderator on which our systematic review focused was students’ identification with STEM.^4^ Briefly, students who identify with a domain see that domain as an important part of who they are, such that their sense of self-worth is to some extent linked with their outcomes in that domain (Boaler, 2002; Cobb et al., 2009; Martin, 2000; Steele, 1997). Whether or not a student has identified with STEM may matter because students with differing levels of STEM identification tend to have different concerns in the STEM classroom (Cheryan & Plaut, 2010; Easterbrook & Hadden, 2021; Steele, 1997). In particular, students who have not yet identified with STEM are typically less concerned with negative stereotypes about their ability and are more concerned with belonging than students who have already identified with STEM domains (Cheryan & Plaut, 2010; Drury et al., 2011; Steele, 1997). As a result, students who have not identified with STEM might be more effectively motivated by role models who foster a sense of belonging (that is, of being accepted and valued by others) in STEM, whereas students who have already identified with STEM might benefit most from exposure to role models who can decrease concerns about negative stereotypes, allowing them to maintain and strengthen their identification with STEM.

### Distinctive theoretical and empirical contributions of the present research

Before describing our methods and findings, we differentiate the present systematic review from two recent articles that have also sought to summarize segments of the evidence on role models: a 2018 qualitative review by Olsson and Martiny and a 2019 meta-analysis by Lawner and colleagues. How does the present work build on these previous reviews to make a distinctive contribution?

First, Olsson and Martiny (2018) and Lawner et al. (2019) did not structure their reviews to investigate which specific features of a role model explain their effects on students’ motivation. Rather, these previous reviews focused on aggregate categories such as counterstereotypical role models (Olsson & Martiny, 2018) or ingroup role models (Lawner et al., 2019), which are undoubtedly important but may also miss systematic variability in the effectiveness of a role model. Here, we unpacked these categories into specific features or dimensions that can more precisely identify the reasons why role models are effective or ineffective. For instance, an ingroup role model may be either motivating or demotivating depending on the level of competence they project and whether their success seems attainable to the students.

Second, we extended what was previously reviewed to include a broader range of students and role models. Olsson and Martiny (2018) focused their review on the effects of role model exposure on girls and women, whereas we included a broader range of students. Lawner et al. (2019) focused on ingroup role models—that is, role models who share students’ group membership—whereas we included results obtained with outgroup role models as well. The breadth along these two dimensions (students and role models) was essential considering that our guiding question is, “Which role models are effective for which students?”.

Third, we anchored our review in the education research on motivation because this research has the potential to be informative about the psychological mechanisms that underlie role models’ effects. By situating our review in the motivation literature, we sought to provide a theoretical framework that can adequately explain when role models motivate (or fail to motivate) and maximize the relevance and usefulness of our review for the education research and practice communities.

A fourth and final distinctive feature of our review is that we distilled a summary of take-aways from the role model literature for use by researchers or practitioners who wish to implement role model interventions in their local contexts. Given the enduring enthusiasm about role models as a tool for diversifying STEM, it is important to extract digestible recommendations from this unwieldy literature. Formulating such recommendations, even if they are tentative, is preferable to the current state of affairs, in which most educators are forced to rely on their intuitions about what works when introducing their students to role models.

### The present systematic review

To summarize, our goal here was to provide a systematic review of the literature on role models in STEM in order to answer the key question of which role models are effective for which students. The answer to this question is likely to provide an accessible tool for diversifying STEM. Our review of this literature was organized in terms of three role model moderators (the role model’s perceived competence, their similarity to the student, and the attainability of their STEM career) and four student moderators (gender, race/ethnicity, age, and identification with STEM). We then used this review to formulate practical recommendations for maximizing the benefits of exposing diverse students to STEM role models.

The choice of a systematic review, rather than a traditional narrative review or a meta-analysis, was motivated by two considerations. First, unlike traditional narrative reviews, systematic reviews are characterized by a “methodical, replicable, and transparent approach” (Siddaway et al., 2019, p. 749). The methodical, comprehensive nature of systematic reviews lowers the likelihood of bias in selecting and summarizing research, and their transparently reported methodology (e.g., search terms, inclusion criteria) facilitates reproducibility by enabling other researchers to verify the authors’ conclusions. Second, a meta-analysis was not feasible here because our key research goal—namely, identifying which role models are effective for which students—led us to include studies whose methodologies (in terms of research design, outcome variables, etc.) were too diverse for quantitative summarization via meta-analysis.

## Method

Our methodology in conducting this systematic review followed the most recent guidelines provided in the literature (Moher et al., 2015; Siddaway et al., 2019), as we describe next.

### Search process

Multiple databases were searched to obtain relevant research studies: PsycINFO, Psychology and Behavioral Sciences Collection (EBSCO), ERIC, and ProQuest Dissertations and Theses. The following search terms were used in this electronic database search: (“role model” or “role models”) AND (math* OR science OR “STEM”). These electronic databases were searched through August 13, 2019, and 1562 articles were generated from the electronic database search.

Because researchers use a variety of terms to refer to role models (e.g., Lawner et al., 2019), in a second step, we broadened our search to include other, related terms: (“role model” or “role models” OR ingroup mentor OR ingroup expert OR ingroup peer) AND (math* OR science OR “STEM”). Although our definition of role models explicitly differentiates them from mentors, not everyone follows this terminological distinction, so we included the term “mentor” in this broader list to identify relevant research that simply used different terminology. We found 14 additional articles through this broader search. Any of these articles that did not match our definition of a role model (e.g., because the STEM expert had a prior relationship with the students) were excluded during the screening process, as detailed below.

We additionally performed a search on Google Scholar using the two sets of search terms above to ensure we were not missing any articles that may not have come up in the other databases. We found 13 additional articles to include in the screening process through this search.

### Screening process

To be included, studies had to (a) expose participants to a role model, (b) be conducted in a STEM-relevant domain (e.g., math), and (c) include STEM-specific outcome variables (e.g., performance, interest, sense of belonging). In addition, (d) the full text had to be available and in English. We did not make random assignment an inclusion criterion because this would have excluded a large number of field studies and program evaluations that did not randomly assign participants to conditions. However, following the definition of a role model articulated above, (e) we excluded any studies that examined parents, teachers, or other individuals with whom students were personally acquainted as role models.

We first screened the titles and abstracts of the articles according to these inclusion/exclusion criteria. However, due to the limited information provided in abstracts and titles, articles were excluded at this stage only if the given information was sufficient to clearly conclude that a criterion was not fulfilled (e.g., the research did not concern a STEM domain). This process narrowed down the list of potential articles to 394 records.

**Fig. 1:**
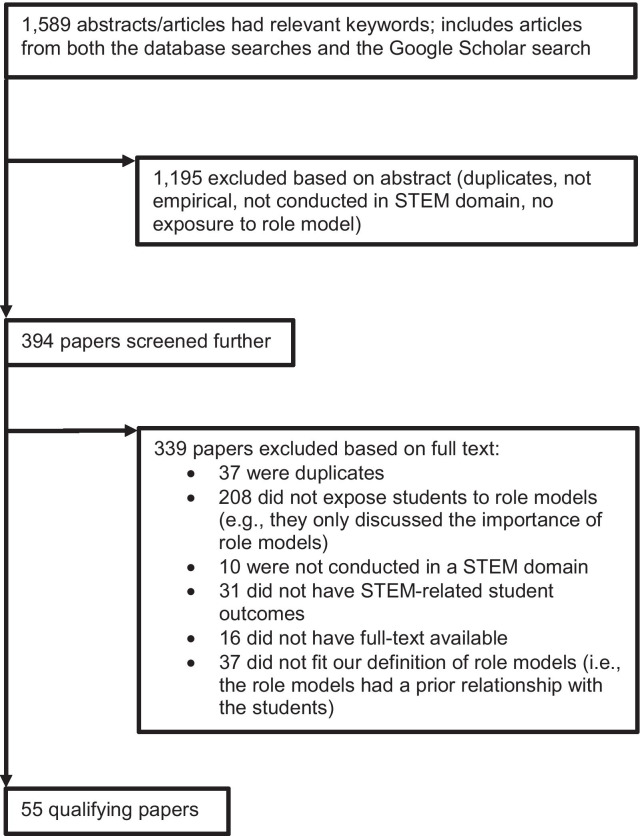
Flowchart of the steps in the screening process

After eliminating duplicates (*n* = 37), a set of 357 articles were subjected to full-text screening. The first author and two trained research assistants went through these texts and determined whether they met the inclusion criteria (inter-rater agreement = 94%; disagreements were resolved via discussion). Articles were excluded because participants were not exposed to a role model (*n* = 208), because the studies were not conducted in a STEM domain (*n* = 10), because no STEM-specific student outcome was assessed (*n* = 31), because the full text was not available (*n* = 16), or because the STEM expert had a prior relationship with students (*n* = 37). After this last screening step, the first author and the research assistants agreed that 55 articles met the inclusion criteria (see Fig. 1 for screening flowchart). A complete list of the articles included is provided in Additional file 1.

### Data extraction and synthesis process

For each of the moderators of interest, two research assistants (the same as those involved in inclusion/exclusion coding) and the first author examined the 55 articles in the final sample to extract relevant results. The articles relevant to a moderator were divided into those that pertained to a single level of the moderator (e.g., a high level of role model competence) vs. those that compared across levels of the moderator (e.g., high vs. low levels of role model competence). Studies that provide comparisons are more informative about the effects of a moderator and were thus privileged at the synthesis stage. The three researchers worked independently and then came together to arrive at a consensus for each moderator via discussion, in consultation with the second author. The first author then synthesized the output of this data extraction process into a narrative summary that the second author double-checked for accuracy against the original text of the articles and revised accordingly.

### Additional methodological considerations

Implementing the data extraction and synthesis process above led us to omit from consideration an additional moderator that we had initially intended to examine: the type of outcome measured. Given the wide range of motivation-related outcomes measured in the literature on role models, especially relative to the somewhat modest size of this literature, it was not feasible to draw robust conclusions about the effects of specific role model features (e.g., competence) on specific outcome variables such as self-efficacy or interest.^5^ Thus, we use the umbrella term “motivation” to synthesize the results of the articles in our target sample, except in cases where a more specific term was useful. Notably, we also included achievement outcomes (e.g., grades, test performance) under this umbrella. Although achievement and motivation are distinct constructs, motivational variables reliably influence achievement (e.g., Eccles & Wigfield, 2020; Schunk & Usher, 2019; Yeager et al., 2019), so it is likely that role model features that affect motivation will also affect achievement.

In addition, we made an a priori decision not to formally assess study quality as a moderator. Although still common, the practice of scoring study quality has come under increasing scrutiny because of a general lack of agreement on which elements of a study speak to its quality (e.g., Sanderson et al., 2007) and—more fundamentally—on what is even meant by “quality” (e.g., Valentine & Cooper, 2005), as well as because of evidence that existing tools for measuring study quality are not valid (e.g., Jüni et al., 1999). Instead of using a formal measure of study quality, we followed Siddaway et al.’s (2019) recommendation to adopt a presentation style that includes information about the sample and methods whenever possible, which enables readers to form their own conclusions about the quality of the evidence being synthesized.

## Results and discussion

We organize our synthesis of the findings into two sections. First, we discuss evidence pertinent to role model moderators—that is, features of role models that might increase vs. decrease students’ STEM motivation—focusing in particular on the three features identified through our analysis of prominent theories of motivation: role models’ competence, their similarity to the students, and the attainability of their STEM career. Second, we discuss evidence pertinent to student moderators, focusing in particular on students’ gender, race/ethnicity, age, and their identification with STEM. Table 1 summarizes the evidence described in more detail below.

**Table 1:** Summary of role model moderators and student moderators

Moderator	Relationship to role model’s effectiveness
*Role model moderators*	
1) Perceived competence	The relationship between the perceived competence of the role model and *ingroup* students’ motivation had an inverted-U shape: Describing the role model as competent increased student motivation and performance, but only up to a point. When a role model’s competence was exceptional, the role model was often demotivating. This moderator either had no consistent effects on *outgroup* students or showed a reverse pattern whereby low-competence models had positive effects
2) Perceived similarity to students	
2a) Demographic similarity	When the role models belonged to groups that are underrepresented in STEM (e.g., women, Black people), they often had positive effects for *all* students, regardless of demographic similarity. In contrast, majority-group models (e.g., men, White people) did not motivate students from underrepresented groups and were sometimes demotivating
2b) Psychological similarity	Characteristics that increased the role model’s psychological similarity to students (e.g., widely shared characteristics such as a preference for spending time with close others; characteristics that contradicted common stereotypes of scientists) generally had positive effects on student motivation. Prompting students to reflect on their similarity to the role models was also sometimes effective
3) Success is perceived as attainable	Role model features and behaviors that increased the perceived attainability of the role model’s success (e.g., “demystifying” STEM careers for students by describing the responsibilities that come with such jobs and the concrete steps to pursue them) were positively related to student motivation. Notably, every instance in which exposure to a role model backfired, lowering STEM motivation (vs. increasing it or having no effect), was linked in some way to the perceived unattainability of the role model’s career
*Student moderators*	
1) Gender	The range of role models that were motivating for students from groups that are underrepresented in STEM (in particular, girls and racial/ethnic minorities) was narrower than that for majority-group students, and the probability of backfire effects was also greater among underrepresented-group students
2) Race/ethnicity	
3) Age	There was no difference in the extent to which role models were effective as a function of student age
4) Identification with STEM	The prediction was that a general sense of similarity to people in STEM, regardless of whether this similarity is demographic or psychological, might be sufficient to attract students to STEM in the first place, whereas at subsequent stages (once students have identified with STEM) demographic similarity to role models might take on particular importance. There was not enough evidence to confidently evaluate this prediction

The summaries above gloss over some of the nuances that are presented in the text

Throughout, our discussion of each moderator is often limited to evidence on its “main effects” (e.g., does the perceived competence of the role model make a difference?). We acknowledge, however, that these moderators likely interact with each other in complex ways. We were occasionally able to incorporate this interactive complexity into our synthesis of the literature, especially when relating role model moderators to student moderators—an important part of answering the question of which role models are effective for which students. Nevertheless, there are many other interactive relationships among these moderators that we do not discuss because they have not yet been investigated in the literature. Thus, the present summary of the effects of role models as tools for diversifying STEM is by necessity (over)simplified.

### Role model moderators

#### Competence

Most articles in our sample presented students with a successful, competent STEM role model—unsurprisingly, since being successful is a key reason why someone would be considered a role model. For our purposes, however, the most informative studies are those in which role models of varying competence levels are presented; without variability in this feature, we cannot assess its relationship to student motivation. The relevant studies suggest that this relationship likely has an inverted-U shape: Describing the role model as competent is likely to increase student motivation and performance—especially if other features are in place as well, such as a certain degree of similarity between the student and role model—but only up to a point. When a role model’s competence is described as exceptional, exposure to the role model can backfire and demotivate (see also Lockwood & Kunda, 1997).

Illustrating the positive slope of the inverted-U function, several studies suggested that exposure to an ingroup role model who is portrayed as competent (vs. of ambiguous competence) has beneficial effects on students’ motivation and performance (Buunk et al., 2007; Marx & Roman, 2002; Marx et al., 2013; McIntyre et al., 2005, 2011), particularly when the role model’s background is also similar to students’ own backgrounds (e.g., grew up in the same place, enjoyed the same types of activities; Marx & Ko, 2012).

Evidence for the negative slope of the inverted-U (at more extreme levels of role model competence) is relatively sparse but suggestive. Woodcock (2012) found that exposure to female STEM role models described as “exemplary students with exceptional grades” (p. 21) decreased identification with math (measured implicitly, but not explicitly; Study 2) and math performance (Study 3) among female undergraduates compared to exposure to a role model of average competence. Similarly, Ziegler and Stoeger (2008) found that exposure to a female role model who was “extremely” competent (p. 512) decreased confidence and interest in STEM among girls with low (but not high) baseline levels of interest in STEM compared to exposure to a role model that was “discernibly untalented” (p. 509).

We caution that our claim of an inverted-U-shaped relationship between role model competence and student motivation in STEM is extrapolated from a (small) set of studies that were quite different in terms of their design, dependent variables, etc. This claim should thus be viewed as tentative until further empirical tests are performed. It is also important to keep in mind that most of the studies reviewed in this section focused on the effects of role models’ competence on ingroup students (e.g., female students exposed to a female role model). The few that also examined outgroup students (e.g., male students exposed to a female role model) suggest a different pattern of results—either no consistent effects of the outgroup role model (e.g., Ziegler & Stoeger, 2008; male participants in Study 1 of Marx and Roman (2002)) or a reverse pattern whereby performance is facilitated at low levels of role model competence (e.g., Study 2 of Marx and Roman (2002)). These results serve as a reminder that the features of a role model often act in tandem to influence student motivation, with the effects of one feature (e.g., competence) sometimes varying as a function of the values of another (e.g., similarity).

#### Similarity to students

We now synthesize the results pertaining to whether the STEM role model’s similarity to students is motivating. Two distinct types of similarity between role models and students emerged in our review (e.g., Eby et al., 2013; Hernandez et al., 2017b). The first is demographic similarity. A role model is demographically similar to a student if the two share a social identity—most important for our purposes here, gender or race/ethnicity. The second type of similarity is psychological. A role model is psychologically similar to a student to the extent that the two share certain preferences, values, struggles, etc., regardless of whether they also share any social identities. While the two types of similarity are correlated, they also have distinct relationships with student motivation. We discuss each in turn.

*Demographic similarity.* Anecdotally, it is often assumed that a role model must be demographically similar to students in order to motivate them. For instance, the slogan “you can’t be what you can’t see” presupposes a match of (visible) social identities between the role model and the student. While our review is operating with a broader notion of role models, the literature nevertheless reflects this narrower view: Many articles in our sample simply matched participants and role models in their demographics—specifically, gender and/or race/ethnicity (Bamberger, 2014; Betz & Sekaquaptewa, 2012; Davis, 2001; Dubetz & Wilson, 2013; Ferreira, 2001; Fox, 1976; Gilbert, 2015; Goldberg & Sedlacek, 1995; Hammrich et al., 1999; Herrmann et al., 2016; Holmes et al., 2012; Johnson, 1989; Kant et al., 2018; Liu et al., 2014; Mbano & Nolan, 2017; Murray et al., 2009; O’Brien et al., 2017; Phelan et al., 2017; Rosenthal et al., 2013; Schriver et al., 1995; Scott, 2013; Swindell & Phelps, 1991). Most of these studies used versions of a pre- vs. post-test design to assess the effects of exposure to demographically similar role models on students’ motivation for STEM, finding a preponderance of positive results on outcomes such as confidence, interest, identity, course-taking intentions, and achievement (for exceptions, see Bamberger, 2014; Betz & Sekaquaptewa, 2012; Holmes et al., 2012).

However, as in the preceding section, in the absence of a comparison (demographically similar vs. dissimilar role model), it is difficult to attribute these positive effects to the similarity between the role model and the students. Thus, we next looked to the subset of studies in our sample that varied the demographic match between role models and students and that also reported the results separately for the students who matched vs. mismatched the role models’ demographics.^6^

This set of studies revealed an interesting asymmetry in the breadth of role models’ positive effects depending on their demographics: When the role models belonged to groups that are underrepresented in STEM (such as women or Black people), they often had broad positive effects on students, regardless of demographic match vs. mismatch between the role model and the students (Bagès & Martinot, 2011; Conner & Danielson, 2016; Evans et al., 1995; Plant et al., 2009; Smith & Erb, 1986; Ziegler & Stoeger, 2008; for an exception, see Hoffman & Kurtz-Costes, 2019). For instance, Evans et al. (1995) found that exposure to female role models improved attitudes toward science among girls and (to a lesser extent) boys in a sample of 964 ninth-grade students. Conner and Danielson (2016) reported similar findings in a sample of 231 students in grades 2–6, except that some of their effects were actually stronger for boys than for girls. In a smaller number of studies, underrepresented-group STEM role models had a positive effect on ingroup students, but no effect on the outgroup (i.e., majority-group) students (McIntyre et al., 2005; Stout et al., 2011)—or the models’ effects on majority-group students were not measured (Johnson et al., 2019; Shapiro et al., 2013). Notably, a “bonus feature” of underrepresented-group STEM role models is that—beyond their effects on students’ own motivation—they also seem to reduce students’ stereotypes against marginalized minorities in STEM (Plant et al., 2009; Smith & Erb, 1986).

Although STEM role models from underrepresented groups often motivate majority-group students, the converse is not true: In many of the studies that reported the relevant comparisons, majority-group STEM role models (e.g., men, White people) did not motivate students from underrepresented groups (Dennehy & Dasgupta, 2017; Shapiro et al., 2013; Stout et al., 2011) and were sometimes demotivating (Marx & Roman, 2002; Stout et al., 2011). Exposure to majority-group role models had broader positive effects only in a restricted range of circumstances: In particular, these broader effects emerged when the majority-group models displayed additional features or behaviors that increased their similarity and appeal to students—for instance, when they were young, attractive, and “cool” (Plant et al., 2009), when they were friendly toward students (e.g., nodding, smiling; Krämer et al., 2016), when their success was explained as a function of malleable factors such as their efforts (Bagès et al., 2016), or when they contradicted the stereotype of STEM professionals as “nerdy loners” (e.g., the models reported liking to play sports rather than video games; Cheryan et al., 2011, 2013). We return to this issue in the section on psychological similarity.

It may be that majority-group STEM role models are less-than-effective motivators for marginalized students because they highlight the stereotypes that make this domain unwelcoming for students like them (e.g., that STEM is a masculine domain). In contrast, underrepresented-group STEM role models may not activate similar threats in majority-group students, who are generally unlikely to question their place in STEM and might therefore see the role model simply as an inspiring adult whose success they can try to emulate.

This asymmetry in the role of demographic similarity, whereby role models belonging to groups that are traditionally underrepresented in STEM are more broadly motivating (regardless of their demographic similarity to students) than majority-group models, has important practical implications: Because most classrooms consist of students with a mix of social identities and backgrounds, one might reasonably worry that exposing an entire classroom to a role model (e.g., a female scientist) might demotivate the subset of students who do not share that role model’s social identities (e.g., the boys). In light of this research, it seems that exposure to role models from underrepresented groups avoids this potential issue and is likely to result in a net motivation gain on average while also not being discouraging to any subsets of students.

*Psychological similarity.* Students can feel similar to a STEM role model—and thus be inspired by the role model’s success—not just because they are part of the same group but also because they perceive the role model to be “like them” at a deeper, psychological level. Our analysis of the articles in our sample revealed two distinct strategies for leveraging psychological similarity to increase the effectiveness of role models. The first strategy targets the role models, highlighting a subset of their psychological characteristics that students may be likely to identify as similar to their own. The second strategy targets the students, asking them to reflect on the role models’ characteristics and identify similarities to themselves. We discuss each of these strategies in turn. We should also note that, as before, only some of the articles included a comparison between the effects of role models in the presence vs. absence of strategies to leverage psychological similarity; we mark these articles, which are particularly informative, with an asterisk in the two subsections that follow.

*Strategy targeting the role models.* Our review revealed that the role model characteristics that were highlighted for students tended to fall at the intersection of two categories. First, the highlighted role model characteristics tended to be ones that most people share (e.g., a preference for spending time with close others rather than alone). Highlighting this category of broadly shared characteristics is potentially beneficial because it maximizes the probability that students will perceive the role models as similar to themselves from a psychological standpoint. Second, the role model characteristics that were highlighted for students tended to be ones that contradicted common stereotypes of scientists (e.g., the stereotype that they prefer solitude and are innately brilliant; Boston & Cimpian, 2018; Cheryan et al., 2015). Highlighting this category of characteristics is potentially beneficial because it corrects students’ mistaken assumptions about scientists, portraying scientists as more psychologically similar to the “average person” than students might have otherwise assumed them to be.

Specifically, the studies in our sample highlighted two role model characteristics that fell at the intersection of these categories, both of which had positive effects on student motivation: (1) STEM role models’ preference to be around and help other people (Cheryan et al., 2011*, 2013*; Marx & Ko, 2012*; Plant et al., 2009; Tan-Wilson & Stamp, 2015), a preference that is common in the general population and stereotypically assumed to be rare among scientists, and (2) STEM role models’ history of working hard and persisting in the face of failure (Bagès & Martinot, 2011*; Bagès et al., 2016*; Hernandez et al., 2017a; Herrmann et al., 2016; Hong & Lin-Siegler, 2012*; Lin-Siegler et al., 2016*; Shin et al., 2016), which—again—is common in students’ lives and stereotypically assumed to be rare in scientists’ lives (given their supposed brilliance).

A couple of caveats are in order here. First, Lawner (2014*) is an exception to the pattern that psychological similarity to a role model boosts STEM motivation; Lawner’s experimental studies found no effect of messages that STEM role models work with and help others. Second, some of these studies included other cues that could have increased role models’ psychological similarity to the students, beyond their other-oriented preferences and the hard work they put in to succeed. These cues included hobbies and other leisure preferences (e.g., reading *Rolling Stone* magazine rather than *Electronic Gaming Monthly*; Cheryan et al., 2011*, 2013*) and aspects of the role models’ personal histories intended to match the students’ own (e.g., growing up in the same region; Marx & Ko, 2012*). Thus, while we are confident in the conclusion that psychological similarity is a reliable moderator of role models’ effects, the specific (combinations of) features that are sufficient to induce this sense of similarity is a matter that deserves further investigation.

*Strategy targeting the students.* The goal of increasing the perceived psychological similarity between role models and students can be achieved not only by highlighting select characteristics of the role model (as described in the preceding section) but also by prompting the students to reflect on the ways in which they are similar to the role models. Indeed, the experimental studies that adopted versions of this strategy generally found positive effects on students’ STEM attitudes (Gilbert, 2015; O’Brien et al., 2017*; Van Camp et al., 2019*; for an exception, see Hoffman & Kurtz-Costes, 2019). Arguably, role model- and student-focused strategies could be combined as well, which might further enhance their effectiveness in promoting STEM motivation.

#### Attainable success

Is perceiving a role model’s success as attainable (i.e., within reach) motivating for students? Because the features that determine a role model’s motivational effects are interrelated, several patterns of results described in previous sections are relevant to understanding the role of attainability as well. First, the inverted-U-shaped relationship between a role model’s competence and their ability to motivate students is relevant here because extreme levels of competence are also less attainable. Second, the findings concerning the importance of the perceived similarity (demographic and psychological) between role models and students are relevant here because the career of a role model who seems similar to the self may thereby also appear more attainable. Thus, these two sets of findings can be reasonably interpreted as providing evidence for the claim that the attainability of a role model’s success is positively related to student motivation.

A distinct dimension of attainability identified in our review pertains to role models’ ability to fill in informational gaps about the wide range of STEM careers that exist, the specific responsibilities that come with such jobs, and the concrete steps that a student might take to pursue them. Studies in which role models provided such information, thus making the possibility of a STEM career less hazy and more attainable, seemed to generally benefit students’ motivation to pursue STEM (Hughes et al., 2013; Johnson, 1989; Kant et al., 2018; Mbano & Nolan, 2017; Mills & Katzman, 2015; Newbill, 2005; Phelan et al., 2017; Wyss et al., 2012).

Finally, we also note that, in the articles in our sample, every instance in which exposure to a role model backfired—lowering STEM motivation (vs. increasing it or having no effect)—was linked in some way to the perceived unattainability of the role model’s career. There are many reasons why role models’ success can appear to students to be too difficult to match. In addition to the examples already discussed that involved extreme competence (Woodcock, 2012; Ziegler & Stoeger, 2008), Bamberger (2014) reported that their sample of Israeli ninth-grade girls were “frightened” (p. 557) by their interactions with female scientists and engineers from a nearby technology company, perhaps in part because the role models used complex scientific concepts that the students were unfamiliar with or because they described some of the challenges involved in working in a male-dominated environment (which may have made their jobs seem undesirable in addition to unattainable). In contrast, the backfire effect reported by Betz and Sekaquaptewa (2012) in their sample of US middle-school girls had a different source: Female STEM role models were demotivating when they also displayed stereotypically feminine features (e.g., wearing pink clothes and makeup).^7^ The success of a feminine-presenting woman in a domain that is both male-dominated and (stereotypically) unfeminine may have seemed too difficult to emulate. Considering the elevated risk that accompanies this role model moderator, it seems important to carefully calibrate the extent to which a role model’s success will appear attainable to students in any future interventions, regardless of scale.

### Student moderators

We now discuss whether role models’ effectiveness varies as a function of four student characteristics. We first focus on students’ (1) gender and (2) racial/ethnic background as potential moderators of role models’ effects, in part because these demographic dimensions are particularly relevant to the goal of making STEM more diverse. Next, we address the question of whether role models may be differentially effective for (3) students of different ages and for (4) students who have vs. have not identified with STEM as a domain.

#### Gender and race/ethnicity

Our ability to assess the moderating effects of student gender and race/ethnicity was constrained by the fact that 69% of studies in our sample included as participants only members of groups that are underrepresented in STEM—in particular, most of these studies included only girls or women, and the rest included only Black and/or Latinx students. Although this is a reasonable design decision considering that a key goal of this literature is to understand how to increase the representation of these groups in STEM, it does make it more difficult to discern whether, and in what ways, role models are differentially effective depending on students’ demographics.

Even so, aggregating across the subset of papers that reported separate results by gender and race/ethnicity, an important conclusion emerges—the flip side of the earlier conclusion about the breadth of role models’ effects as a function of their demographics: The range of role models that are motivating for students from groups that are underrepresented in STEM is narrower than that for majority-group students, and the probability of backfire effects is also greater among underrepresented-group students.

This difference can be seen most clearly when examining the effects of outgroup role models. While outgroup role models are usually motivating for majority-group students (e.g., Bagès & Martinot, 2011; Conner & Danielson, 2016), their effects on underrepresented-group students are much more mixed (Johnson et al., 2019; Marx & Roman, 2002; Shapiro et al., 2013; Stout et al., 2011). This is not to say that outgroup models *cannot* motivate students from underrepresented groups—the bar is simply higher, in that outgroup role models need to display some additional features that increase their relatability (e.g., Bagès et al., 2016; Cheryan et al., 2011, 2013; Krämer et al., 2016). We also are not claiming that *ingroup* role models are always motivating for students from underrepresented groups. In fact, in the preceding section, we discussed several striking instances where exposure to female role models demotivated girls (e.g., Bamberger, 2014; Betz & Sekaquaptewa, 2012; see also Hoffman & Kurtz-Costes, 2019). Note, however, that no studies in our sample reported that exposure to an ingroup model demotivated majority-group students.^8^ Thus, these additional datapoints are consistent with the claim that the set of circumstances under which role models are motivating for underrepresented-group students is smaller (and, conversely, that the set of circumstances under which role models are demotivating for these students is larger). This pattern of findings is not unexpected considering the reality of belonging to a group that is underrepresented and stigmatized in STEM contexts: For these students, the range of day-to-day STEM environments where they feel psychologically “safe” is narrower (e.g., Schmader & Hall, 2014), and the findings of our systematic review likely reflect this simple but powerful fact.

#### Age

Among the studies in our sample, 9% focused on elementary school students, 25% focused on middle school students, 24% focused on high school students, and 42% focused on college students. Thus, the literature is skewed toward older students, and studies on elementary school students are relatively rare.

There was no noticeable difference in the extent to which role models were effective as a function of student age. Role models were as likely to motivate elementary school students as they were to motivate undergraduates. This conclusion is consistent with prior findings, in that Lawner and colleagues’ (2019) meta-analysis did not find evidence of moderation by student age either.

However, the conclusion that age is not a moderator of role models’ effects is also qualified by several limitations of the evidence. Most critically, almost all studies included only a narrow age range and thus could not compare the effectiveness of role models for students of different ages. Only two studies reported age/grade comparisons (Conner & Danielson, 2016; Wyss et al., 2012), and neither found age differences. Although these two null effects are consistent with our overall conclusion of no moderation by age, the possibility remains that this conclusion is confounded by methodological differences between studies with younger vs. older students. For example, as many as 80% of the studies with elementary school students exposed students to the role models “live” (i.e., in person), whereas only 25% of the studies with college students did so. In addition, more of the studies with younger children were broader interventions to boost STEM motivation that included elements other than role models (e.g., hands-on activities). In contrast, more of the studies on college studies were lab-based experiments that manipulated only aspects of students’ exposure to role models. It is also noteworthy that role models were generally closer in age (i.e., more demographically similar) to the participants in studies with college students (which always used other adults as role models) than in studies with younger students (which also used mostly adults as role models).

In summary, the data provided qualified support for the conclusion that student age does not moderate the effectiveness of STEM role models as motivational tools. However, our review also revealed gaps in the evidence needed to establish this conclusion with a high degree of confidence. In particular, more work is needed that conducts closely matched, within-study comparisons of the effects of role models on students of different ages.

#### Identification with STEM

As a reminder, the rationale for looking at identification with STEM as a moderator of the effects of role models is that students with differing levels of identification with STEM have different concerns in the classroom and may therefore be looking to role models to fulfill different psychological needs. According to prominent arguments (e.g., Drury et al., 2011), a salient question for students who are not (yet) identified with STEM is whether they fit in with people in this field (e.g., Bian et al., 2018; Cheryan & Plaut, 2010). As a result, role models should be able to boost motivation to pursue STEM among these students to the extent that they lead students to anticipate fitting in and belonging. In principle, a broad range of role models should be able to convey this sense of belonging in STEM, not just ones that are demographically similar to the students (i.e., from the same social group) but also ones that are only similar at a psychological level, in the sense of sharing students’ own goals, preferences, values, etc. In contrast, once a student has identified with STEM and decided to pursue a career in this domain, the question that rises to the fore is whether they will be able to succeed. For students from groups that are underrepresented in STEM, the negative stereotypes that target their groups are a particular source of concern, so these students may derive unique benefits from exposure to demographically similar role models, who have succeeded in spite of these stereotypes. To summarize, this argument predicts that a general sense of similarity to people in STEM, regardless of whether this similarity is demographic or psychological, might be sufficient to attract students to STEM in the first place, whereas at subsequent stages, demographic similarity to successful role models might take on particular importance—especially for students from underrepresented groups, who have to contend with negative perceptions about their groups.

A direct test of this argument would involve exposing students who are high vs. low in STEM identification to role models who are high vs. low in demographic similarity to the students and, orthogonally, high vs. low in psychological similarity to the students. As far as we know, this study has not been conducted—at the very least, it was not in our analytic sample. However, some of the studies examined here provide partial support for this prediction. For instance, several studies on non-STEM-identified students suggested that, for these students, psychological similarity to a role model was at least as motivating as demographic similarity (Bagès & Martinot, 2011; Bagès et al., 2016; Cheryan et al., 2011, 2013; Hong & Lin-Siegler, 2012), consistent with the argument that providing a general sense of fitting in is often sufficient at the recruitment stage. Also consistent with this argument, studies that focused on STEM-identified students highlighted the benefits of exposure to demographically similar role models (Marx & Roman, 2002; Stout et al., 2011).

On the other hand, we did not find any evidence that demographic similarity is particularly important for STEM-identified students, largely because the relevant comparisons were absent in our sample (and likely in the literature more generally). Somewhat relevant, one study that included STEM-identified students suggested that psychological similarity continues to be beneficial, above and beyond demographic similarity, even at this later stage (Marx & Ko, 2012). However, in this study, students’ STEM identification was analyzed as a covariate rather than a moderator, so the finding reported technically pertains to students with average (rather than high) levels of STEM identification. The take-away here is that, despite a few promising hints, much more research is needed before we can confidently describe how students’ identification with STEM moderates the effects of exposure to role models.

## How do we maximize the motivating effects of STEM role models? Four tentative recommendations

**Fig. 2:**
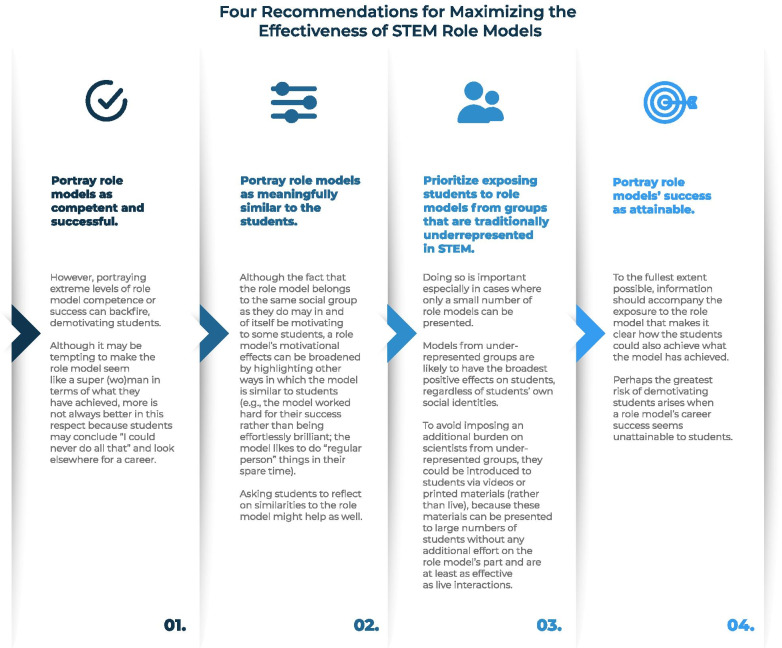
Four recommendations to consider when exposing students to role models as a means of boosting their STEM motivation. High-resolution versions of this infographic in .eps, .jpg, .pdf, and .png formats are freely available at https://doi.org/10.6084/m9.figshare.c.5681674.v1

Based on our synthesis of the evidence (see Table 1 for a summary), we formulated a set of concrete recommendations that the education research and practice community can consider when exposing students from diverse backgrounds to role models as a means of boosting their STEM motivation. Certainly, the evidence base for these recommendations could be stronger; as we have pointed out throughout, many questions still await systematic investigation. However, given that the enthusiasm for role model interventions among educators and the general public continues to run ahead of the research, the benefits of formulating a set of recommendations on the evidence so far, limited as it is, outweigh the potential drawbacks. To enable readers to share these recommendations with others, we have created an infographic (see Fig. 2) that we have also made available in several high-resolution formats via Figshare: https://doi.org/10.6084/m9.figshare.c.5681674.v1.

First, we recommend that role models are portrayed as being competent and successful. The preponderance of the evidence reviewed suggested that these features are motivating in a role model. However, portraying extreme levels of role model competence or success can backfire, demotivating students. Although it may be tempting to make the role model seem like a super(wo)man in terms of what they have achieved, more is not always better in this respect because students may conclude “I could never do all that” and look elsewhere for a career.

Second, we recommend that role models are portrayed as being meaningfully similar to the students. Although the fact that a role model belongs to the same social group as they do may in and of itself be motivating to some students, the role model’s motivational effects can be broadened by highlighting other ways in which the role model is similar to students (e.g., the model worked hard for their success rather than being effortlessly brilliant; the model likes to do “regular person” things in their spare time). Asking students to reflect on similarities to the role model might help as well.

Third, we recommend prioritizing exposure to role models who belong to groups that are traditionally underrepresented in STEM, especially in cases where only a small number of role models can be presented. Role models from underrepresented groups are likely to have the broadest positive effects on students, regardless of students’ own social identities. To avoid imposing an additional burden on scientists from underrepresented groups, they could be introduced to students via videos or printed materials (rather than live) because these materials can be presented to large numbers of students without any additional effort on the role model’s part and are at least as effective as live interactions (Lawner et al., 2019).

Fourth, we recommend that role models’ success is portrayed as attainable. To the fullest extent possible, information should accompany the exposure to the role model that makes it clear how the students could also achieve what the role model has achieved. Perhaps the greatest risk of demotivating students arises when a role model’s career success seems unattainable to students.

## Limitations and future directions

The conclusions of the present systematic review are somewhat tentative, in part because the evidential base is still limited. This literature may also suffer from publication bias. For example, Lawner and colleagues’ (2019) meta-analysis found that lab studies from this literature that had small sample sizes produced larger effects than expected, suggesting a file-drawer problem. In addition, we caution that some of the studies in our target sample (especially those conducted outside the lab) included enrichment activities beyond exposure to role models. Discerning the separate benefits of role models vs. these other activities is impossible when interpreting the results of these studies.

Another limitation of the present systematic review is that we considered motivation- and achievement-related outcomes together rather than separately. The decision to do so was motivated both by practical considerations—the sparsity of the research on each moderator meant that we could not feasibly analyze the data separately by outcome—and by theoretical considerations, insofar as motivation is a causal antecedent of achievement (e.g., Eccles & Wigfield, 2020; Schunk & Usher, 2019; Yeager et al., 2019). In addition, Lawner and colleagues’ (2019) meta-analysis found that interest (a motivational variable) and performance were affected to similar degrees by exposure to role models, which suggests that combining these outcomes is defensible. However, motivation is not the only antecedent of achievement, so it is possible that certain role model or student moderators affect motivation and performance differently. Future research on this topic would be valuable.

Relatively few of the studies we identified through our search focused on students from racial/ethnic minority groups that are underrepresented in STEM. None focused on students with disabilities or on students who identify as lesbian, gay, bisexual, transgender, or queer (LGBTQ), groups that are also underrepresented in STEM careers (e.g., Freeman, 2018; National Science Foundation, 2017). Broadening the scope of research in this literature to include overlooked social identities would be beneficial. Greater attention to the question of effective role models for students who embody multiple identities that are underrepresented in STEM is needed as well. Only one of the 55 articles in our sample focused on this important issue (Johnson et al., 2019).

At a more conceptual level, we caution that focusing on role models as a solution for increasing diversity in STEM risks sending the message that diversifying STEM is simply a matter of motivating students to pursue it. The problem with this message is that it overlooks the systemic biases present in STEM contexts (e.g., racism, sexism, heterosexism, ableism), which make it difficult for some students to succeed regardless of how motivated or capable they are. Although it may be intuitively appealing and cost-effective to intervene by exposing students to role models, this strategy can only succeed if it is part of a broader set of measures to make STEM more welcoming to *all* students who might choose it as a career path.

## Conclusion

The idea of inspiring students—particularly female and racial/ethnic minority students—to pursue STEM by exposing them to role models is ever popular. Here, we systematically reviewed the literature on this topic to identify strategies for maximizing the motivational impact of role models: *Which* role models are effective for *which* students? Our findings, which we distilled into four simple take-aways, provide a bird’s-eye view of the literature for researchers and a practical guide for any educators and policymakers who might want to implement role-model interventions in their local contexts. Our review also identified open questions about why and for whom role models are motivating, questions that we hope will guide future research on this topic.

## Supplementary Information

**Additional file 1.** Complete list of articles included in the systematic review.

## Data Availability

This is a systematic review of prior literature. All articles we reviewed here are either openly available via Google Scholar or accessible through a library subscription. A complete list of the papers included in the systematic review is provided in the supplementary online materials. The infographic summarizing our recommendations for maximizing the effectiveness of role models in STEM (see Fig. 2) is available at https://doi.org/10.6084/m9.figshare.c.5681674.v1.

## Abbreviations

LGBTQ: Lesbian, gay, bisexual, transgender, or queer
STEM: Science, technology, engineering, and mathematics

## Footnotes

1 What matters for student motivation is not whether a role model objectively possesses a certain feature such as competence but rather whether students subjectively perceive the model to possess that feature. Thus, our use of the term “role model features” in this section (and throughout the paper) is intended to stand in for “perceived role model features.”.

2 This argument pertains to attainment and utility value most directly (Eccles and Wigfield, 2020). The term “attainment value” refers to perceived importance of an activity that is somehow tied to a student’s sense of self and to their identity (e.g., a student who has identified with STEM is likely to place high attainment value on this domain). “Utility value” refers to the perceived usefulness of a particular activity for a student’s goals, regardless of whether these goals reflect the student’s deeper self/identity (e.g., a student who wants a job in STEM because STEM careers are lucrative is likely to place high utility value on taking advanced math courses).

3 However, mindset theorists and attributional theorists disagree in how they conceptualize ability per se (e.g., Graham, 2020): Attributional theorists typically view ability as stable and uncontrollable (akin to aptitude), whereas mindset theory is premised on the existence of multiple perspectives on ability (as stable and uncontrollable [fixed mindset] vs. unstable and controllable [growth mindset]).

4 This variable is correlated with age: Students who have identified with STEM are likely to be older on average than students who have not. However, STEM identification is also meaningfully distinct from age, not just statistically (in that one can find STEM-identified and non-STEM-identified students in any age group) but also conceptually (in that STEM identification is a psychological, attitudinal variable, whereas age is a demographic one).

5 We also note that previous attempts to draw such distinctions between outcomes did not find that outcomes were differentially sensitive to role model exposure (Lawner et al., 2019).

6 Of course, students can match the role model’s demographics on one dimension (e.g., gender) and mismatch them on another dimension (e.g., race). With a single exception (Johnson et al., 2019), no studies in our sample explicitly examined the effects of a match vs. mismatch along multiple social identities. Johnson and colleagues found that Black women students who were exposed to a Black woman or a Black man as a role model had greater feelings of belonging in STEM compared to Black women students who saw either a White woman or White man as a role model; there was no difference between the two White role models. In this instance, it appears that a demographic match along the race/ethnicity dimension mattered more for Black women students’ motivation than a match along the gender dimension. We return to the issue of how few studies examine multiple social identities in the later section on limitations.

7 This effect was only present among girls who did not identify with STEM.

8 It is important to keep in mind, however, that fewer studies included majority-group students in the first place.
