# Supplementary material for: Which role models are effective for which students? A systematic review and four recommendations for maximizing the effectiveness of role models in STEM
Source: Int J STEM Educ. 2021 Dec 2;8(1):59. doi: 10.1186/s40594-021-00315-x (PMC8636406; doi:10.1186/s40594-021-00315-x)
Supplement: Supplementary file 1 — Additional file 1. Complete list of articles included in the systematic review. [file 40594_2021_315_MOESM1_ESM.pdf]

ADDITIONAL ONLINE MATERIALS

Which Role Models are Effective for Which Students? A Systematic Review and Four Recommendations for Maximizing the Effectiveness of Role Models in STEM

Jessica R. Gladstone and Andrei Cimpian

Complete List of Articles Included in the Systematic Review

Bagès, C., & Martinot, D. (2011). What is the best model for girls and boys faced with a standardized mathematics evaluation situation: A hard-working role model or a gifted role model? *British Journal of Social Psychology, 50,* 536–543. http://dx.doi.org/10.1111/j.2044-8309.2010.02017.x

Bagès, C., Verniers, C., & Martinot, D. (2016). Virtues of a hardworking role model to improve girls' mathematics performance. *Psychology of Women Quarterly, 40,* 55–64. https://doi.org/10.1177/0361684315608842

Bamberger, Y. M. (2014). Encouraging girls into science and technology with feminine role model: Does this work? *Journal of Science Education and Technology*, *23,* 549–561. https://www.jstor.org/stable/24019742

Betz, D. E., & Sekaquaptewa, D. (2012). My fair physicist? Feminine math and science role models demotivate young girls. *Social Psychological and Personality Science*, *3*, 738–746. https://doi.org/10.1177/1948550612440735

Buunk, A. P., Peiró, J. M., & Griffioen, C. (2007). A positive role model may stimulate career-oriented behavior. *Journal of Applied Social Psychology*, *37,* 1489–1500. https://doi.org/10.1111/j.1559-1816.2007.00223.x

Cheryan, S., Drury, B. J., & Vichayapai, M. (2013). Enduring influence of stereotypical computer science role models on women's academic aspirations. *Psychology of Women Quarterly*, *37*(1), 72–79. https://doi.org/10.1177/0361684312459328

Cheryan, S., Siy, J. O., Vichayapai, M., Drury, B. J., & Kim, S. (2011). Do female and male role

models who embody stem stereotypes hinder women's anticipated success in stem? *Social Psychological and Personality Science*, *2*, 656–664. https://doi.org/10.1177/1948550611405218

Conner, L. D. C., & Danielson, J. (2016). Scientist role models in the classroom: how important is gender matching? *International Journal of Science Education*, *38*(15), 2414–2430. https://doi.org/10.1080/09500693.2016.1246780

Davis, N. J. (2001). *A study of the impact of a science symposium designed to influence middle school females toward further studies in science.* Ph.D., The University of Iowa. ProQuest Dissertations and Theses (3018568).

Dennehy, T. C., & Dasgupta, N. (2017). Female peer mentors early in college increase women's positive academic experiences and retention in engineering. *Proceedings of the National Academy of Sciences*, *114*, 5964–5969. https://doi.org/10.1073/pnas.1613117114

Dubetz, T., & Wilson, J. A. (2013). Girls in engineering, mathematics and science, GEMS: A science outreach program for middle-school female students. *Journal of STEM Education, 14(3)*, 41–47. Google Scholar

Evans, M. A., Whigham, M., & Wang, M. C. (1995). The effect of a role model project upon the attitudes of ninth-grade science students. *Journal of Research in Science Teaching, 32*(2), 195–204. Google Scholar

Ferreira, M. (2001). Building communities through role models, mentors, and hands-on-science. *School Community Journal, 11*(2), 27–38. Google Scholar

Fox, L. H. (1976). *Changing behaviors and attitudes of gifted girls.* Paper presented at the Annual Meeting of the American Psychological Association, Washington, DC.

Gilbert, P. (2015). *The role of role models: How does identification with STEM role models impact women's implicit STEM stereotypes and STEM outcomes?* Ph.D., Tulane University. *ProQuest Dissertations and Theses* (3703388).

Goldberg, J. L. & Sedlacek, W.E. (1995) *Summer Study in Engineering for High School Women.* (ERIC Document Reproduction Service No. ED 399 876). Google Scholar

Hammrich, P. L., Richardson, G. M., & Livingston, B. (1999). Sisters in science: An intergenerational science program for elementary school girls. *School Community Journal*, *8*(2), 21–35. Google Scholar

Hernandez, D., Rana, S., Rao, A., & Usselman, M. (2017). Dismantling stereotypes about Latinos in STEM. *Hispanic Journal of Behavioral Sciences*, *39*(4), 436–451. https://doi.org/10.1177/0739986317731100

Herrmann, S. D., Adelman, R. M., Bodford, J. E., Graudejus, O., Okun, M. A., & Kwan, V. S. Y. (2016). The Effects of a Female Role Model on Academic Performance and Persistence of Women in STEM Courses. *Basic and Applied Social Psychology, 38*, 258–268. https://doi.org/10.1080/01973533.2016.1209757

Hoffman, A. J., & Kurtz-Costes, B. (2019). Promoting science motivation in American Indian middle school students: An intervention. *Educational Psychology*, *39*(4), 448–469. https://doi.org/10.1080/01443410.2018.1527019

Holmes, S., Redmond, A., Thomas, J., & High, K. (2012). Girls helping girls: Assessing the influence of college student mentors in an afterschool engineering program. *Mentoring & Tutoring: Partnership in Learning*, *20*(1), 137–150. https://doi.org/10.1080/13611267.2012.645604

Hong, H. Y., & Lin-Siegler, X. (2012). How learning about scientists' struggles influences students' interest and learning in physics. *Journal of Educational Psychology*, *104*(2), 469–484. https://doi.org/10.1037/a0026224

Hughes, R. M., Nzekwe, B., & Molyneaux, K. J. (2013). The single sex debate for girls in science: A comparison between two informal science programs on middle school students' STEM identity formation. *Research in Science Education*, *43*, 1979–2007. https://doi.org/10.1007/s11165-012-9345-7

Johnson, J. (1989). *Effects of successful female role models on young women's attitudes toward traditionally male careers.* Paper presented at the Annual Meeting for the Association for Educational Communications and Technology, Dallas, TX. Google Scholar

Johnson, I. R., Pietri, E. S., Fullilove, F., & Mowrer, S. (2019). Exploring identity-safety cues and allyship among black women students in STEM environments. *Psychology of Women Quarterly*, *43*(2), 131–150. https://doi.org/10.1177/0361684319830926

Kant, J., Burckhard, S. & Meyers, R. (2018). Engaging high school girls in Native American culturally responsive STEAM activities. *Journal of STEM Education*, *18*(5), 15–25. https://www.learntechlib.org/p/182466/

Krämer, N. C., Karacora, B., Lucas, G., Dehghani, M., Rüther, G., & Gratch, J. (2016). Closing the gender gap in STEM with friendly male instructors? On the effects of rapport behavior and gender of a virtual agent in an instructional interaction. *Computers & Education*, *99,* 1–13. https://doi.org/10.1016/j.compedu.2016.04.002

Lawner, E. K. (2014). *Impact of role model gender and communality on college women's math performance and interest in STEM.* Unpublished master's thesis. Storrs, CT: University of Connecticut.

Lin-Siegler, X., Ahn, J. N., Chen, J., Fang, F. F. A., & Luna-Lucero, M. (2016). Even Einstein struggled: Effects of learning about great scientists' struggles on high school students' motivation to learn science. *Journal of Educational Psychology, 108,* 314–328. https://doi.org/10.1037/edu0000092

Liu, Y., Lou, S., & Shih, R. (2014). The investigation of STEM Self-Efficacy and Professional Commitment to Engineering among female high school students. *South African Journal of Education, 34*(2), 1–15. https://doi.org/10.15700/201412071216

Marx, D. M., & Ko, S. J. (2012). Superstars 'like' me: The effect of role model similarity on performance under threat. *European Journal of Social Psychology, 42,* 807–812. https://doi.org/10.1002/ejsp.1907

Marx, D. M., Monroe, A. H., Cole, C. E., & Gilbert, P. N. (2013). No doubt about it: When doubtful role models undermine men's and women's math performance under threat. *The Journal of Social Psychology, 153,* 542–559. https://doi.org/10.1080/00224545.2013.778811

Marx, D. M., & Roman, J. S. (2002). Female role models: Protecting women's math test performance. *Personality and Social Psychology Bulletin, 28,* 1183–1193. http://dx.doi.org/10.1177/01461672022812004

Mbano, N., & Nolan, K. (2017). Increasing access of female students in science, technology, engineering, and Mathematics (STEM), in the University of Malawi (UNIMA). *Science Education International*, *28*(1), 53–77. Google Scholar

McIntyre, R. B., Lord, C. G., Gresky, D. M., Ten Eyck, L. L., Frye, G. D. J., & Bond, C. F., Jr. (2005). A social impact trend in the effects of role models on alleviating women's mathematics stereotype threat. *Current Research in Social Psychology, 10,* 116–137.

Google Scholar

McIntyre, R. B., Paulson, R. M., Taylor, C. A., Morin, A. L., & Lord, C. G. (2011). Effects of role model deservingness on overcoming performance deficits induced by stereotype threat. *European Journal of Social Psychology, 41*(3), 301–311. https://doi.org/10.1002/ejsp.774

Mills, L. A., & Katzman, W. (2015). *Examining the effects of field trips on science identity.* Paper presented at the 12th International Conference on Cognition and Exploratory Learning in Digital Age, Kildare, Ireland. Google Scholar

Murray, N. G., Opuni, K. A., Reininger, B., Sessions, N., Mowry, M. M., & Hobbs, M. (2009). A multi-media educational program that increases science achievement among inner-city non-Asian minority middle-school students. *Academic Medicine, 84*, 803–811. https://doi.org/10.1097/ACM.0b013e3181a425e7

Newbill, P. L. (2005). *Instructional strategies to improve women's attitudes toward science*. Ph.D., Virginia Polytechnic Institute and State University. ProQuest Dissertations and Theses (3164126). Google Scholar

O'Brien, L. T., Hitti, A., Shaffer, E., Camp, A. R. V., Henry, D., & Gilbert, P. N. (2017). Improving girls' sense of fit in science: increasing the impact of role models. *Social Psychological and Personality Science*, *8*(3), 301–309. https://doi.org/10.1177/1948550616671997

Phelan, S. A., Harding, S. M., & Harper-Leatherman, A. S. (2017). BASE (broadening access to science education): A research and mentoring focused summer STEM camp serving underrepresented high school girls. *Journal of STEM Education: Innovations and Research*, *18*(1), 65–72. Google Scholar

Plant, E. A., Baylor, A. L., Doerr, C. E., & Rosenberg-Kima, R. B. (2009). Changing middle school students' attitudes and performance regarding engineering with computer-based social models. *Computers & Education*, *53*(2), 209–215. https://doi.org/10.1016/j.compedu.2009.01.013

Rosenthal, L., Levy, S. R., London, B., Lobel, M., & Bazile, C. (2013). In pursuit of the MD: The impact of role models, identity compatibility, and belonging among undergraduate women. *Sex Roles, 68*(7–8), 464–473. https://doi.org/10.1007/s11199-012-0257-9

Schriver, M. L., Wolfe, L., & Strickland, W. J. (1995, April). *A case study of a science and mathematics day camp as experienced by seven girls from rural Georgia*. Paper presented at the annual meeting of the National Association for Research in Science Teaching, San Francisco, CA. Google Scholar

Scott, J. R. (2013). *Einstein girls: Exploring STEM careers interest and identity in an online mentoring community* (Publication No.3584495) [Doctoral dissertation, University of Florida]. ProQuest Dissertations Publishing. Google Scholar

Shapiro, J. R., Williams, A. M., & Hambarchyan, M. (2013). Are all interventions created equal? A multi-threat approach to tailoring stereotype threat interventions. *Journal of Personality and Social Psychology*, *104*(2), 277–288. http://dx.doi.org/10.1037/a0030461

Shin, J. E. L., Levy, S. R., & London, B. (2016). Effects of role model exposure on STEM and non-STEM student engagement. *Journal of Applied Social Psychology*, *46*, 410–427. https://doi.org/10.1111/jasp.12371

Smith, W. S., & Erb, T. O. (1986). Effect of women science career role models on early adolescents' attitudes toward scientists and women in science. *Journal of Research in Science Teaching*, *23*(8), 667–676. Google Scholar

Stout, J. G., Dasgupta, N., Hunsinger, M., & McManus, M. A. (2011). STEMing the tide: Using ingroup experts to inoculate women's self-concept in science, technology, engineering, and mathematics (STEM). *Journal of Personality and Social Psychology*, *100*, 255–270. http://dx.doi.org/10.1037/a0021385

Swindell, R. & Phelps, M. (1991). *Designing and Implementing Science Enrichment Programs for Rural Females*. Paper presented at the Rural Education Symposium, Nashville, TN. Google Scholar

Tan-Wilson, A., & Stamp, N. (2015). College students' views of work–life balance in STEM research careers: Addressing negative preconceptions. *CBE—Life Sciences Education*, *14*(3), 1–13. https://doi.org/10.1187/cbe.14-11-0210

Van Camp, A. R., Gilbert, P. N., & O'Brien, L. T. (2019). Testing the effects of a role model intervention on women's STEM outcomes. *Social Psychology of Education*, *22*(3), 649–671. https://doi.org/10.1007/s11218-019-09498-2

Woodcock, A. (2012). *Implicit ingroup biases and performance under threat: Do successful*

counterstereotypic exemplars help or harm? (Publication No. 3544586) [Doctoral dissertation, Purdue University]. ProQuest Dissertations Publishing. Google Scholar

Wyss, V. L., Heulskamp, D., & Siebert, C. J. (2012). Increasing middle school student interest in STEM careers with videos of scientists. *International Journal of Environmental and Science Education*, *7*(4), 501–522. Google Scholar

Ziegler, A., & Stoeger, H. (2008). Effects of role models from films on short-term ratings of intent, interest, and self-assessment of ability by high school youth: A study of gender-stereotyped academic subjects. *Psychological Reports*, *102*(2), 509–531. https://doi.org/10.2466/pr0.102.2.509-531
